# The integration window for shape cues is a function of ambient illumination

**DOI:** 10.1186/1744-9081-3-15

**Published:** 2007-03-14

**Authors:** Ernest Greene

**Affiliations:** 1 Laboratory for Neurometric Research, Department of Psychology, University of Southern California, Los Angeles, California 90089-1061, USA; 2 Neuropsychology Foundation, Sun Valley, CA 91353, USA

## Abstract

Minimal discrete shape cues, *i.e.*, dots that marked positions on the outer boundary of namable objects, were divided into two subsets, which were shown very quickly with a variable delay between subsets. Recognition of a given object required integration of the information provided by the two subsets, and previous research had found that recognition declined as the delay between subsets was increased. The present experiment found the decline in recognition to be linear for each of several levels of ambient illumination, dropping rapidly under photopic test conditions, and with the slope being progressively less steep with transition into the scotopic range. The change in the duration of information persistence may be related to the density of information that is provided under various lighting conditions, and a requirement that the information be buffered against noise or "packaged" to accommodate successive saccades.

## Background

"*All the connections set up between sensations by the formation of ideas tend to persist, even when the original conditions of connection are no longer fulfilled*." Titchener [1]

It is well established that brief stimulation can initiate sustained neural activity that allows information to be sampled or integrated over time intervals that far outlast the duration of the stimulus. In vision, the persistence of information has been variously described as visual information store [2], iconic memory [3], and short-term visual storage [4].

**Figure 1:**
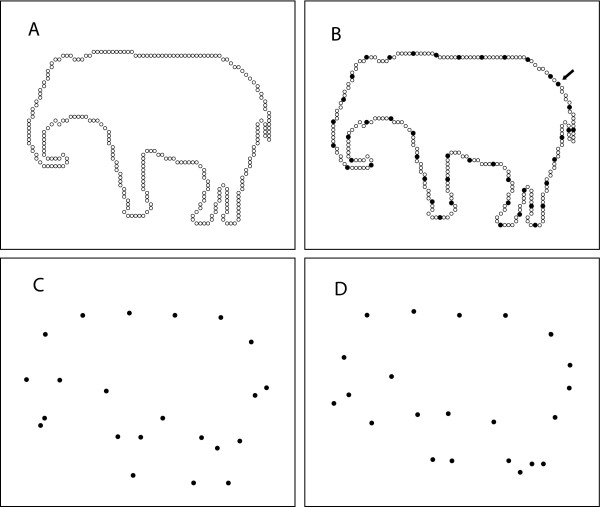
The upper left panel shows the full complement of boundary dots for one of the shapes to be identified. A sampling of these dots is shown in the upper right panel as filled circles, this being an example of a display set. To pick the display set for a given subject, the sampling began at a randomly selected starting point, shown by the arrow, and included every N^th ^dot, counting clockwise from this location. [See text for discussion of how N was determined for each shape.] The display set was then divided into subsets, one containing the odd dots from the counting process, and the other containing the even dots. These are shown in the lower left and right panels, respectively. The dots in each subset were displayed as a group, varying the time interval between each subset as a function of room illumination.

Previous research from this laboratory found that the information persistence needed for recognition of transient discrete shape cues is affected by the level of ambient room illumination [5]. In those experiments, objects were represented using a sparse sampling of dots that marked the outer boundary of each object. Fig. 1 shows an example from that study, which was used also in the present experiment. The upper left panel of Fig. 1 shows the full inventory of dots that specified locations on the outer boundary. A sample was drawn from that inventory for display to a given subject, as illustrated in the upper right panel, and this sample was designated as the "display set." The display set was further divided into subsets, one containing the dots lying at odd positions in the sequence, and the other containing the dots at even positions, as shown in the lower panels of Fig. 1.

Under these test conditions, the prior work found that if the two subsets were displayed with minimal delay between offset of the first subset and onset of the second, recognition levels were relatively high [5]. However, adding a delay between the two subsets impaired recognition of the shapes, and the degree of impairment was a function of ambient light level [5]. One experiment examined the amount of information persistence with normal room lighting versus darkness, and found that recognition levels dropped fairly quickly in the former, but only moderately in the latter even with subset delays of over 200 ms [5]. A second experiment tested in a dim room, and found an intermediate rate of decline, along with evidence that the decrease was a linear function of the delay interval [5].

These results [5] provided evidence for differentials in the persistence of shape-cue information that were a function of light level, but the delay intervals were not optimal for showing the rate of decline at each level of ambient illumination. The present experiment provided a more strategic sampling of time intervals, and has yielded evidence for linear declines having slopes that are a function of this illumination.

## Methods

Ten USC undergraduates served as subjects in the experiment. Subjects had normal or corrected to normal visual acuity. Except for the task instructions described below, they were naive to the hypothesis under consideration. Subjects received course credit for their participation.

The shapes to be identified were taken from the Macmillan Visual Dictionary [6] or from Hemera's clip art [7]. A custom program positioned a 64 × 64 array over the image, requiring that the object span the full dimension of the array in either the vertical or the horizontal direction. Then the cells of the array that fell on the outer boundary of the object were marked, meaning that the column and row position of each boundary location was entered into an address table. To provide a consistent rule for adjacency and basis for specifying distance among marked locations, a requirement was imposed that one could use only a continuous sequence of adjacent cell locations, not allowing inclusion of any cell previously visited.

One hundred fifty (150) shapes were used in the present experiment, as shown in Table 1 (following References). Each shape was displayed to a given subject only once using a minimal transient discrete cue protocol. In this protocol, only some of the dots that mark the boundary of the object are shown, designated as the display set. The number of dots in the display set, and their spacing, was chosen to provide approximate equivalence in potential for recognition (as determined by earlier experiment). As illustrated in Fig. 1, the method for selecting the display set for a given subject began by randomly choosing a starting point and then selecting every Nth dot. The value of N ranged from 3 to 10. For each of the objects, Table 1 lists the value of N (designated as the "skip factor"), as well as the percentage and number of dots in the display set.

**Table 1:** The names of shapes used in both experiments are listed below

Shape #	Shape Name	Perimeter	Area	Skip	Dot%	Dot#
1	alarm clock	276	2588	5	20.29	56
2	anchor	210	664	6	16.67	35
3	angel	240	1486	4	25	60
4	antique car	180	1565	4	25	45
5	antique chair	202	1329	3	33.66	68
6	baboon	316	1398	3	33.54	106
7	baby bottle	147	1228	3	33.33	49
8	badge	160	2060	3	33.75	54
9	banana	180	1385	8	12.78	23
10	bat	156	786	5	20.51	32
11	bear	213	1527	4	25.35	54
12	bee	309	1453	3	33.33	103
13	beetle	269	1345	3	33.46	90
14	bell	156	829	4	25	39
15	binoculars	176	1592	3	33.52	59
16	boot	183	1708	8	12.57	23
17	bottle	143	866	8	12.59	18
18	bowling pin	134	890	5	20.15	27
19	buffalo	238	1688	3	33.61	80
20	bull	302	1270	3	33.44	101
21	burro	359	1426	4	25.07	90
22	butterfly	306	2055	10	10.13	31
23	c clamp	267	952	3	33.33	89
24	camel	282	1956	7	14.54	41
25	candelabra	312	750	4	25	78
26	cap	158	1530	4	25.32	40
27	car	136	834	8	12.5	17
28	cat	248	1532	5	20.16	50
29	chair	217	1857	5	20.28	44
30	chick	177	1252	6	16.95	30
31	cordless drill	240	1835	3	33.33	80
32	christmas tree	190	1423	3	33.68	64
33	coat	271	2365	3	33.58	91
34	coat hanger	160	649	6	16.88	27
35	cow	256	1499	5	20.31	52
36	cowboy boot	189	1864	10	10.05	19
37	dagger	183	748	7	14.75	27
38	deer	353	1354	7	14.45	51
39	desk lamp	223	588	4	25.11	56
40	dinosaur	209	827	4	25.36	53
41	dog	280	1517	6	16.79	47
42	dragonfly	246	1068	3	33.33	82
43	duck	172	1025	6	16.86	29
44	dumbbell	185	1837	5	20	37
45	elephant	261	1761	6	16.86	44
46	fighter jet	240	1154	6	16.67	40
47	fire extinguisher	293	1995	3	33.45	98
48	fire hydrant	184	1145	3	33.7	62
49	fish	198	1364	3	33.33	66
50	flask	144	905	3	33.33	48
51	flower	222	2704	3	33.33	74
52	flying pheasant	229	1235	4	25.33	58
53	fox	245	961	3	33.47	82
54	frog	371	2078	4	25.07	93
55	giraffe	353	1137	8	12.75	45
56	glasses	215	649	5	20	43
57	glove	216	1165	3	33.33	72
58	goose	164	916	5	20.12	33
59	gramophone	230	1687	4	25.22	58
60	guitar	151	760	7	14.57	22
61	gun	171	968	4	25.15	43
62	hammer (ball peen)	156	416	3	33.33	52
63	hammer (claw)	168	506	5	20.24	34
64	hand shovel	144	540	3	33.33	48
65	hat	161	1496	6	16.77	27
66	heart	171	2685	9	11.11	19
67	helmet	161	1912	4	25.47	41
68	hen	218	1190	8	12.84	28
69	hippo	255	1984	3	33.33	85
70	horse	348	1588	5	20.11	70
71	horseshoe	273	1310	9	11.36	31
72	house	207	2647	4	25.12	52
73	humming bird	162	747	9	11.11	18
74	industrial hook	208	1431	4	25	52
75	iron	226	2039	3	33.63	76
76	jack rabbit	245	1686	12	8.57	21
77	kangaroo	246	860	5	20.33	50
78	knife	133	355	6	17.29	23
79	leaf	259	1294	7	14.29	37
80	light bulb	145	1461	7	14.48	21
81	lion	283	1334	5	20.14	57
82	lizard	242	976	6	16.94	41
83	macaw	158	726	4	25.32	40
84	man	249	888	4	25.3	63
85	man's shoe	157	1167	8	12.74	20
86	microscope	288	1003	3	33.33	96
87	monkey	256	892	3	33.59	86
88	moth	257	1642	7	14.4	37
89	motor scooter	228	1214	4	25	57
90	motorcycle	239	1355	7	14.64	35
91	mushroom	187	1504	5	20.32	38
92	music stand	193	592	5	20.21	39
93	ostrich	244	843	9	11.48	28
94	pan	151	1239	3	33.77	51
95	passenger plane	243	1038	6	16.87	41
96	pear	146	1174	7	14.38	21
97	pelican	248	1389	3	33.47	83
98	pepper	156	1068	3	33.33	52
99	piano	298	1844	5	20.13	60
100	pickup	154	790	5	20.13	31
101	pig	220	1357	6	16.82	37
102	pipe	151	503	7	14.57	22
103	pliers	224	517	4	25	56
104	porpoise	168	860	7	14.29	24
105	pot	177	1754	4	25.42	45
106	power boat	199	1262	5	20.1	40
107	propane torch	151	728	3	33.77	51
108	ram	392	1682	3	33.42	131
109	rat	192	785	4	25	48
110	rhino	187	1247	3	33.69	63
111	rifle	135	257	5	20	27
112	rooster	249	1453	6	16.87	42
113	sailboat	210	1008	3	33.33	70
114	saxophone	242	902	6	16.94	41
115	scissors	250	1186	5	20	50
116	sea gull	254	1132	3	33.46	85
117	sea horse	172	626	4	25	43
118	sea lion	202	1675	3	33.66	68
119	shark	185	831	7	14.59	27
120	sheep	232	1587	3	33.62	78
121	ship propeller	262	1665	6	16.79	44
122	shorts	192	2309	5	20.31	39
123	sickle	176	473	3	33.52	59
124	slipper	139	830	7	14.39	20
125	snail	176	989	3	33.52	59
126	snake	173	407	4	25.43	44
127	sock	144	823	6	16.67	24
128	spider	363	1112	3	33.33	121
129	spoon	134	416	4	25.37	34
130	spray bottle	180	1034	3	33.33	60
131	starfish	211	1301	9	11.37	24
132	submarine	147	769	4	25.17	37
133	swordfish	200	593	6	17	34
134	table	289	1357	7	14.53	42
135	table lamp	184	1187	5	20.11	37
136	teapot	185	1930	4	25.41	47
137	teddy bear	238	1571	3	33.61	80
138	telephone	200	2012	6	17	34
139	tiger	236	1031	4	25	59
140	toilet	225	2301	3	33.33	75
141	tractor	238	1864	3	33.61	80
142	trumpet	216	895	3	33.33	72
143	turtle	171	1100	5	20.47	35
144	umbrella	199	1764	6	17.09	34
145	vase	164	1562	6	17.07	28
146	violin	174	800	4	25.29	44
147	windmill	243	1330	4	25.1	61
148	wine glass	234	2091	5	20.09	47
149	wolf	267	1441	4	25.09	67
150	woman's shoe	162	874	6	16.67	27

For each shape, the table also provides the following information: Perimeter: the number of dots in the full inventory of boundary locations ; Area: the number of dots enclosed within the perimeter, and including the perimeter dots; Skip: the skip factor, which specified that every Nth dot would be included in the sample that was shown to a given subject; Dot% and Dot#: the percentage and number of dots that were displayed as a result of applying the skip factor

For the present experiment the display set was then divided into two subsets, each containing roughly half of the dots to be displayed. A convention was applied that numbered the address positions of the display set, specifying each odd position as belonging to one subset, and each even position to the other. These were designated as odd and even subsets, as illustrated in the lower two panels of Fig. 1. As detailed below, each subset was displayed as a group, first the odd subset and then the even subset. Varying the time interval between displays of these subsets was a major variable of the experiment, as described below.

Testing was done in a room that had no windows, and fluorescent tubes housed in standard recessed ceiling fixtures with plastic diffusion panes provided the lighting. The level of ambient illumination from these fixtures was controlled by the addition of opaque occluding panels that were held in channels that were coplanar to the surface of the fixture. Each fixture had two panels, one over each end, which could be slid apart to alter the area of the opening through which light could flow. This provided for control of ambient illumination without any change in color temperature of the light.

Three levels of ambient illumination were used in the experiment, designated as bright, dim and dark. Ambient light levels were measured with a Tektronix J17 photometer, which uses a cosine corrected head having certified calibration. The light readings were taken from the location of the seated subject. Mean illumination was 303 lux for the bright condition, and was 13.3 lux for the dim condition. The lights were turned completely off for the dark condition, and the illumination was functionally zero.

Measures were also taken of the amount of light being reflected from the art-board frame and from the wall surrounding the display board (both of which were the same shade of ivory). When the room was bright, the luminance of these surfaces was 25 Cd/m^2^, and for the dim condition the luminance was 1 Cd/m^2^.

Stimulus shapes were presented using a display board having a 64 × 64 array of LEDs, each of which could be illuminated under control of a computer and microprocessor slave. The GaAlAs LEDs emitted at a wavelength of 660 nm, and had a rise/fall time for emission in the range of 50–100 nanoseconds. Two levels of LED emission were used. With the room bright, the emission level was set to 96 Cd/m^2^. When the room was either dim or dark, the emission was set at 7 Cd/m^2^, the lower level being used because brief flashes that are substantially brighter can produce afterimages.

The display board was attached to a wall at a viewing distance of 3.5 m, and with an elevation above eye level of approximately 10 degrees. At this distance the diameter of each LED was 4.9 arc', center-to-center spacing was 7.4 arc', and the dimensions of the full array, *i.e.*, measured from center-to-center of the outside elements, was 7.7 × 7.7 arc°.

**Figure 2:**
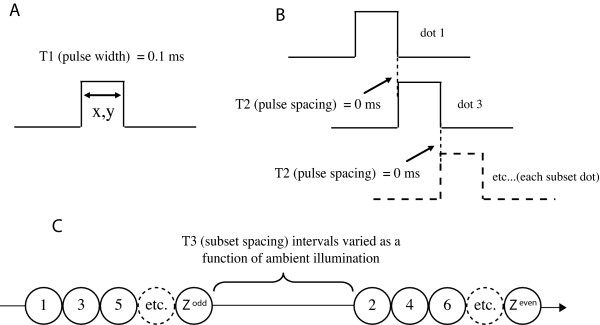
A. The duration that a given LED was illuminated was 0.1 ms. This is designated as T1. B. The dots within a given subset were displayed sequentially with a pulse spacing of 0.1 ms, measured from onset to onset. C. Here the pulse sequences for the odd and even subsets are illustrated like beads on a string. The time required to display a given subset varied with subset size, with the longest interval being 6.6 ms. The temporal separation of the two subsets, designated as T3, varied as a function of room illumination. The ranges for the T3 interval were: bright (0–40 ms); dim (0–80 ms); dark (0–160 ms).

Each dot of the display set was shown on the LED array by allowing current to flow through the specified LED for 0.1 ms, this being designated as T1. It is convenient to describe the display of a given address as a pulse, so T1 specifies pulse width, as illustrated in Fig. 2, this figure having been used in previous work [5].

Figure 2 also shows that the successive members of each subset were displayed with a 0.1 interval between onset of one pulse and onset of the next, this being T2. In other words, each was shown with no temporal separation between offset of a given pulse and onset of the next. Each pulse lasted only 0.1 ms, so a subset containing 20 addresses would be shown in 2 ms. From Table 1 one can see that the number of dots being displayed ranged from 17 (for the car) to 131 (for the ram). This provides a range from the smallest to the largest subset of 8 to 66 dots, thus across all shapes a given subset was displayed in a time that was no less than 0.8 ms, and no more than 6.6 ms.

A major variable of interest was the time interval between subsets, which was measured from offset of the final pulse in the odd subset till onset of the first pulse in the even subset. This was designed as T3. As outlined in the introduction, Greene [5] found a decline of recognition as a function of T3, with the rate of decline being a function of the level of ambient illumination. Therefore, a different range of T3 values was chosen for each level of room illumination, the goal being to sample the range where the greatest decline was likely to be seen.

To be specific, when the room was bright, the T3 intervals were: 0, 10, 20, 30 and 40 ms. When the room was dim, the T3 intervals were: 0, 20, 40, 60 and 80 ms. When the room was dark, these values were: 0, 40, 80, 120 and 160 ms.

The order of room illumination was determined at random for each subject. Subjects were dark adapted for 20 minutes prior to testing with the room being dark.

Shapes that had been assigned to a given level of room illumination were tested as a block, *i.e.*, each was display successively with illumination being the same. For each level of room illumination the order of shape presentation was random, which provided for a random order of T3 values.

Recognition of a given object required integration of shape cues that were provided by the two subsets. Pilot work had shown that the hit rate from display of a single subset would be in the 20% range. Observing hit rates that are substantially above this value provides evidence of the degree to which the shape cues from the two subsets are being combined by the visual system, which may be described as information persistence or iconic memory.

## Results

Previous research had demonstrated that the time interval within which shape information can be integrated shows large differentials as a function of room illumination [5]. The goal of the present research was to provide T3 intervals that would better sample the range over which a given lighting condition would affect recognition.

For a given subject, each shape was displayed only once at one of the fifteen treatment combinations – five levels of T3 interval across three levels of room illumination. The shapes were approximately matched for difficulty level on the basis of the number of dots in the display sample, and the response variable was successful recognition (yes/no).

**Figure 3:**
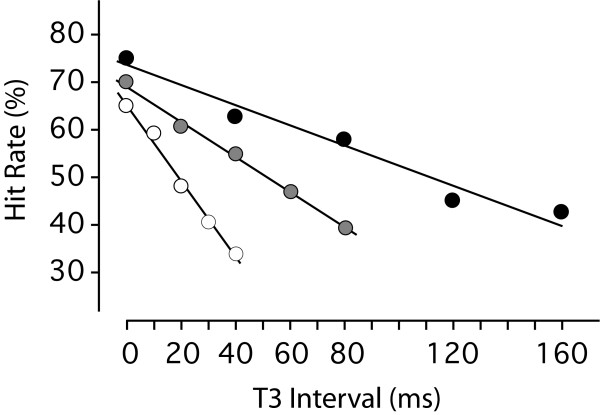
Mean percent recognition (hit rate) dropped at a steep rate in the bright room (open circles), at a moderate rate in the dim room (gray filled circles), and at a relatively shallow rate in the dark room (black filled circles). Statistical modeling showed the decline to be significant at p < .001 for each condition, and there was no indication of departure from the linear regression lines. These results indicate that the information from the odd and even subsets can combine to allow for recognition over longer periods as room illumination is reduced.

Mean recognition level across subjects (hit rate) for each of the fifteen treatment combinations are plotted in Fig. 3, and a linear regression line has been fit to the data for each level of room illumination. At T3 = 0 the hit rates for the bright, dim and dark conditions were 65, 70 and 76 percent, respectively, which depart only moderately from the 75% hit rate that was expected for displays having no temporal separation. From these initial levels, the plots for the three conditions show linear declines, having slopes that were progressively less steep with bright, dim and dark room illumination, respectively.

For statistical confirmation of effects, the appropriate model for this binary data is a generalized linear model with binominal errors [8]. Dot percentage and T3 interval were fixed effects, and subjects and shape were random effects. A separate model was fit to the data from each room illumination condition, since (by design) the ranges of T3 intervals were not comparable. Logit values, *i.e.*, log_e _(proportion/1 – proportion), were calculated, and treatment differences were compared using the standard error of the difference for these values.

For each of the three levels of room illumination, there was a significant decline in the hit rate (p < .001 for each). There was no significant turning point in the response for any level of ambient illumination, *i.e.*, no quadratic effect, with the largest probability being 0.54. This indicates that the decline in recognition is completely linear over the intervals tested for each of the room illumination conditions. Dot percentage was not a significant factor for any of the three models, with the largest probability being 0.32. This indicates substantial success in rendering the shapes to be equivalent in their level of difficulty. Note that proper variance measures for the data are only possible using the logit scores, which precludes the use of error bars on the hit-rate means that are shown in Fig. 3. However, standard errors of the mean can be provided for the logit transformed values, and these are shown in Table 2, along with predictions of hit rate that are provided by the models.

**Table 2:** For each treatment combination, the mean logit score and the standard error of the mean is shown.

T3 (ms)	Light		Dim		Dark	
	Mean	SEM	Mean	SEM	Mean	SEM

0	0.617 (0.650)	0.261	0.958 (0.723)	0.302	1.229 (0.774)	0.271
10	0.382 (0.594)	0.256				
20	-0.107 (0.473)	0.255	0.481 (0.618)	0.292		
30	-0.384 (0.405)	0.257				
40	-0.822 (0.305)	0.264	0.180 (0.545)	0.291	0.660 (0.659)	0.248
60			-0.154 (0.462)	0.289		
80			-0.688 (0.335)	0.297	0.345 (0.585)	0.246
120					-0.173 (0.457)	0.244
160					-0.388 (0.404)	0.244

The logit scores provided the basis for statistical modeling of the data, which found a significant (p < .001) decline in hit rate for each of the room illumination conditions. These models also provide predictions of hit rate, i.e., the values that fall on the linear regression line, for each of the T3 values within the sampled range. These are shown in parentheses beneath each mean logit value. These predictions are very similar to the observed hit rates that are shown in Fig. 3.

In the previous study [5] the level of shape recognition in a bright room appeared to be nearly asymptotic at 35–40% with T3 intervals in the 90–270 ms range. Thus the 35% hit rate observed here with the room bright and with T3 equal to 40 ms may be at or near the floor level. However, the earlier study [5] found that dark room recognition remained at or above 60% with T3 intervals of 90 and 270 ms, whereas the present study found a hit rate of 43% with a T3 of 160 ms. The present study differed from the previous [5] protocol only in the use of an expanded inventory of shapes, and in sampling a more restricted range of T3 intervals. Thus there is no obvious basis for this difference for the dark-room condition. In any event, the earlier result raises the possibility that recognition rates will asymptote at T3 intervals that are longer than those tested here, and the floor level may be progressively higher for bright, dim and dark levels of room illumination.

## Discussion

Prior research from this laboratory [5] used spaced dots to mark the outer boundary of namable objects. For a given object (shape) the dots were divided into two subsets, and were displayed with various intervals of delay between the first and the second subset. Successful recognition of shapes was a function of the duration of this delay, and also of the ambient level of illumination, being shorter when the room was bright, longer in a dim room, and longer yet when the room was completely dark. The present work confirms these effects, and we can now specify that each level of room illumination provides a range in which an increase in subset interval will produce a linear decline in recognition. Recognition was found to be fairly equivalent in the 65–75% range irrespective of ambient light level when the subset interval was zero. From there the increase in subset interval produced linear declines, dropping recognition into the 35–45% range with subset intervals of 40 ms in the bright room, 80 ms in the dim room, and 160 ms when the room was dark.

It is possible that the interval over which information persists, *i.e.*, information persistence, is determined by the level of ambient illumination. It is well understood that the visual system dramatically increases its sensitivity under low-light conditions, and for threshold detection, stimuli are integrated over a longer interval [9-12]. Visible persistence, *i.e.*, the duration over which a very brief stimulus is subjectively perceived [13-16], is also affected by the level of ambient illumination. Di Lollo & Bischof [17] review this relationship and cite twelve studies that have reported changes in integration time as a function of ambient illumination, these effects being attributed to visible persistence. However, Coltheart [14], among others, has argued that information persistence – the integration of information over time – may be mediated by perceptual mechanisms other than visible persistence. The prior work from this laboratory [5] examined whether the information persistence required for object recognition could be explained by the duration of visible persistence, and found that the two manifestations of persistence had different time courses. It appears that the neural mechanisms that provide for the subjective judgment of stimulus duration are not the same as those that allow for integration of successive shape cues.

As an alternative to the concept that information persists for a fixed amount of time that is a function of ambient illumination, it is possible that the interval over which information can be combined is closely tied to the density of the information being provided. In this model, information from a given moment would be "compartmentalized" and buffered against interference from noise and/or incompatible information. Thus with photopic levels of illumination, where large amounts of information are being delivered, the temporal compartment would be relatively short. The compartment interval would become wider as ambient illumination declined, given that the lower illumination also decreased the density of the information being provided at any given moment, as well as the potential for interference. The ability to set the width of the temporal compartment as a function of information density would be especially useful for animals that are highly mobile or move their eyes, as these actions drastically change the image content being provided to the retina from one moment to the next.

Stimulus events that occurred at the same moment would be included in a given temporal compartment. It may be relevant, therefore, that another study from this laboratory [18] has found that the degree of simultaneity in the presentation of border dots determines the percentage of shapes that can be identified. Lack of simultaneity in the millisecond and even submillisecond range produces a significant linear decline in recognition.

A few studies have examined the question of whether the complexity of the information to be processed affects integration time, most being done using a visible persistence protocol of one kind or another. Loftus & Hanna [19], for example, randomly divided visual stimuli into two halves that were presented successively. The stimuli were judged to be most "complete" if there was minimal delay between each half, and progressively less complete with increasing temporal separation. They found that simple dot patterns were affected more at a given delay interval than were complex scenes, suggesting longer persistence of the information contained in the complex scene. Thus, to the extent that one wishes to consider the subjective judgment of "completeness" to be an indication of information persistence, these results are opposite of what would be predicted by the "information density" hypothesis suggested above.

Similar results have been reported by Erwin & Herschenson [20], who assessed the duration of visible persistence by having subjects adjust the onset time of a second stimulus to the perceived offset time of a first stimulus. They evaluated three kinds of stimuli – a blank field, a dark field, and a field containing seven letters. They found that the field of letters persisted about 35 ms longer than the other two stimulus sets if the subjects were required to report the letters. A follow-up study [21] found that the degree of redundancy (and thus complexity) of the letter strings affected the duration of persistence.

Conversely, Irwin & Yeomans [22] argue against the concept that the width of the integration window is a function of the amount of information to be processed. They used a task developed by Hogben & Di Lollo [23] wherein stimulus elements are positioned within a 5 × 5 matrix, displaying a first subset of 12 elements at random positions within the matrix, followed at a variable interval by a second subset of 12 elements. The task is to report which position of the matrix has been left empty, which essentially reflects the duration of visible persistence of the first subset. Irwin & Yeomans [22] conducted five experiments using this protocol, manipulating the degree of stimulus complexity, *e.g.*, letters vs. Xs; upright letters vs. inverted letters, and failed to find any effect of complexity on the duration of visible persistence. They argue that the tasks used by Loftus & Hanna [19] and by Erwin [20,21] assessed cognitive processing operations rather than persistence of the stimulus trace, *per se*.

Prior results from this laboratory [5] found that the interval for integration of shape cues is not related to the duration of visible persistence. It would not be surprising, therefore, if differences in information density provided by various levels of illumination affected shape recognition in a manner that differed from its influence on visible persistence. But additionally, it should be said that the hypothesis relating the integration interval to the density of information pertains to the totality of information provided by the scene. The studies of how complexity of stimuli affects duration of visible persistence [19-22] were not manipulating ambient illumination, and the differentials in stimulus complexity, *e.g.*, upright letters vs. inverted letters, would not produce much net change in the abundance of data being delivered by the entire visual scene.

## Conclusion

Whether one views the process as a change in duration of information persistence, or as compartmentalizing stimulus elements as a function of information density, the present results confirm that there is a change in the duration over which partial shape cues can be combined as one transitions from photopic to scotopic viewing conditions. Additionally, we now know that percent recognition is a linear function of the interval between cue subsets, with a slope that is a function of room illumination. The range for this linear decline is relatively short when the room is bright, and becomes progressively longer with decreasing room illumination.

## List of Abbreviations

arc° : degrees of visual angle

arc' : minutes of visual angle

Cd/m^2 ^: candela per meter squared

GaAlAs : gallium, aluminum and arsenic

LED : light emitting diode

Log_e _: natural log

m : meters

ms : milliseconds

N : number used to specify which dots from address list will be displayed

nm : nanometers

ns : nanoseconds

p : probability

T1 : pulse width

T2 : temporal separation within a given subset

T3 : temporal separation between subsets

## Competing interests

The author declares that he has no competing interests.
